# The Impact of Temperature on Mortality in Tianjin, China: A Case-Crossover Design with a Distributed Lag Nonlinear Model

**DOI:** 10.1289/ehp.1103598

**Published:** 2011-08-09

**Authors:** Yuming Guo, Adrian G Barnett, Xiaochuan Pan, Weiwei Yu, Shilu Tong

**Affiliations:** 1 School of Public Health, and; 2 Institute of Health and Biomedical Innovation, Queensland University of Technology, Brisbane, Australia; 3 School of Public Health, Peking University, Beijing, China

**Keywords:** cardiovascular mortality, case-crossover, distributed lag nonlinear model, mortality, respiratory mortality, temperature

## Abstract

Background: Although interest in assessing the impacts of temperature on mortality has increased, few studies have used a case-crossover design to examine nonlinear and distributed lag effects of temperature on mortality. Additionally, little evidence is available on the temperature–mortality relationship in China or on what temperature measure is the best predictor of mortality.

Objectives: Our objectives were to use a distributed lag nonlinear model (DLNM) as a part of case-crossover design to examine the nonlinear and distributed lag effects of temperature on mortality in Tianjin, China and to explore which temperature measure is the best predictor of mortality.

Methods: We applied the DLNM to a case-crossover design to assess the nonlinear and delayed effects of temperatures (maximum, mean, and minimum) on deaths (nonaccidental, cardiopulmonary, cardiovascular, and respiratory).

Results: A U-shaped relationship was found consistently between temperature and mortality. Cold effects (i.e., significantly increased mortality associated with low temperatures) were delayed by 3 days and persisted for 10 days. Hot effects (i.e., significantly increased mortality associated with high temperatures) were acute and lasted for 3 days and were followed by mortality displacement for nonaccidental, cardiopulmonary, and cardiovascular deaths. Mean temperature was a better predictor of mortality (based on model fit) than maximum or minimum temperature.

Conclusions: In Tianjin, extreme cold and hot temperatures increased the risk of mortality. The effects of cold last longer than the effects of heat. Combining the DLNM and the case-crossover design allows the case-crossover design to flexibly estimate the nonlinear and delayed effects of temperature (or air pollution) while controlling for season.

Heat-related mortality has become a matter of increasing public health significance, especially in the light of climate change. Studies have examined hot and cold temperatures in relation to total nonaccidental deaths and cause-specific deaths (Stafoggia et al. 2006). The city- or region-specific temperature–mortality relationship is often V-, U-, or J-shaped, with increases in mortality at temperatures below the cold threshold or above the hot threshold (Hajat and Kosatky 2010). The temperature–mortality relationship varies greatly by geographic, climate, and population characteristics (Group E 1997). Social, economic, demographic, and infrastructure factors can influence the sensitivity of populations to temperature (Ebi et al. 2006). In China, only a few studies on temperature–mortality relationship have been conducted in Shanghai (Kan et al. 2003), Hong Kong (Chan et al. 2010), and Beijing (Liu et al. 2011). No research has been undertaken in Tianjin, one of the largest cities in northeastern China.

A previous study found that no temperature measure (maximum, mean, or minimum temperature) was consistently better at predicting mortality in the United States. The best temperature measure differed by age group, season, and region (Barnett et al. 2010). It is unknown which temperature measure is the best predictor of mortality in Tianjin.

Mortality risk depends not only on exposure to the current day’s temperature, but also on several previous days’ exposure (Anderson and Bell 2009). The distributed lag model has been applied to explore the delayed effect of temperature on mortality (Analitis et al. 2008; Baccini et al. 2008; Hajat et al. 2005). To overcome the strong correlation between daily temperatures over short time periods, constrained distributed lag structures are used in time-series regressions (Armstrong 2006). The estimates are constrained by smoothing, using methods such as natural cubic splines, polynomials, or stratified lag. Both unconstrained and constrained distributed lag models assume a linear relationship between temperatures below the cold threshold (or above the hot threshold) and mortality, so these models may not be sufficiently flexible to capture the effects of temperature on mortality.

Recently, a distributed lag nonlinear model (DLNM) was developed to simultaneously estimate the nonlinear and delayed effects of temperature (or air pollution) on mortality or morbidity (Armstrong 2006; Gasparrini et al. 2010). DLNMs use a cross-basis function that describes a two-dimensional temperature–response relationship along the dimensions of temperature and lag. The choice of cross-basis functions for the temperature and lag are independent, so the spline or linear functions can be used for temperature, whereas the polynomial functions can be used for the lag. The estimates can be plotted using a three-dimensional graph to show the relative risks along both temperature and lags. We can predict the relative risks for a certain temperature or lag by extracting a slice from the three-dimensional graph. We can compute the overall effect by summing the log relative risks of each lag. Separate smoothing functions are applied to time to control for season and secular trends.

The case-crossover design controls for seasonal effects and secular trends by matching case and control days in relatively small time windows (e.g., calendar month). This controls for season using a step function rather than a smooth spline function (Barnett and Dobson 2010). Most previous studies used the case-crossover design with relatively inflexible models to investigate the effects of temperature on mortality, such as assuming a linear effect for temperature in each season, with a single lag model, or moving average lag model (Basu et al. 2008; Green et al. 2010). Few studies have demonstrated how to fit nonlinear and delayed effects of temperature on mortality within a case-crossover design.

We used DLNMs combined with the case-crossover design, making it possible to fit more sophisticated estimates of the effects of temperature (or air pollution) using a case-crossover design. We demonstrated these models here using a motivating example of the temperature–mortality relationship in Tianjin, China, and also investigated which temperature measure had the best predictive ability for mortality.

## Materials and Methods

*Data collection.* Tianjin is a northeastern Chinese city adjacent to Beijing and Hebei Province and located along the coast of Bohai Gulf (39° 07´ north, 117° 12´ east). Tianjin has four distinct seasons, with cold, windy, dry winters (due to vast Siberian anticyclones) and hot, humid summers (due to monsoons). It is the fifth-largest Chinese city in terms of urban land area. The population in the urban area was 4.2 million in 2005.

Mortality data were obtained from the China Information System for Death Register and the Report of Chinese Centre for Disease Control and Prevention from 1 January 2005 to 31 December 2007. The mortality data were from six urban districts of Tianjin: Heping, Hedong, Hexi, Nankai, Hebei, and Hongqiao. We classified nonaccidental mortality according to the *International Classification of Diseases, 10th revision* (ICD-10 codes A00–R99; World Health Organization 2007). Cardiopulmonary (ICD-10 codes I00–I99 and ICD-10 codes J00–J99), cardiovascular mortality (ICD-10 codes I00–I99), and respiratory mortality (ICD-10 codes J00–J99) were examined separately.

Daily meteorological data on maximum, mean, and minimum temperature and relative humidity were obtained from the China Meteorological Data Sharing Service System (http://cdc.cma.gov.cn). Daily air pollution data on particulate matter < 10 μm in aerodynamic diameter (PM_10_), sulfur dioxide (SO_2_), and nitrogen dioxide (NO_2_) were obtained from the Tianjin Environmental Monitoring Centre (Tianjin, China).

*Data analysis.* We used the time-stratified case-crossover with a fixed and disjointed window (e.g., calendar month), avoiding the overlap bias (Janes et al. 2005). The case-crossover using conditional logistic regression is a special case of time-series analysis (Lu and Zeger 2007). This equivalence provides computational convenience and permits model checking for the case-crossover design using standard log-linear model diagnostics (Lu et al. 2008). We used a Poisson regression model that allows for overdispersion to combine a DLNM with the case-crossover design:

*Y_t_ ~* Poisson(*μ_t_*) *Log* (*μ_t_*) = α + β*T_t,l_* + *S*(*RH_t_*, 3) + *S*(*PM_10t_*, 3) + *S*(*SO_2t_*, 3) + *S*(*NO_2t_*, 3)+ λStrata*_t_* + ηDOW*_t_* + υHoliday*_t_* + δInfluenza*_t_* = α + β*T_t,l_* + *COVs*, [1]

where *t* is the day of the observation; *Y_t_* is the observed daily death counts on day *t*; α is the intercept; *T_t,l_* is a matrix obtained by applying the DLNM to temperature, β is vector of coefficients for *T_t,l_*, and *l* is the lag days. *S(…)* is a natural cubic spline. Three degrees of freedom (df) were used to smooth relative humidity and PM_10_, NO_2_, and SO_2_ concentrations in accordance with previous studies (Anderson and Bell 2009; Stafoggia et al. 2008). Strata*_t_* is a categorical variable of the year and calendar month used to control for season and trends, and λ is vector of coefficients. DOW*_t_* is day of the week on day *t*, and η is vector of coefficients. Holiday*_t_* is a binary variable that is “1” if day *t* was a holiday, and υ is the coefficient. Influenza*_t_* is a binary variable that is “1” if there were any influenza deaths on day *t,* and δ is the coefficient. *COVs* represents all other covariates in the model.

Based on the vector of estimated coefficients β in Equation 1, the DLNM was used to predict the effects and standard errors for combinations of temperature and lags. Graphs, summaries, and statistical inference can be obtained from DLNM estimates and standard errors (Armstrong 2006).

We used a natural cubic spline–natural cubic spline DLNM that modeled both the nonlinear temperature effect and the lagged effect using a natural cubic spline. We placed spline knots at equal spaces in the temperature range to allow enough flexibility in the two ends of temperature distribution. We placed spline knots at equal intervals in the log scale of lags to allow more flexible lag effects at shorter delays. To completely capture the overall temperature effect and adjust for any potential harvesting (i.e., heat-related excesses of mortality were followed by deficits), we used lags up to 27 days according to a previous study (Armstrong 2006). The median value of temperature was defined as the baseline temperature (centering value) for calculating the relative risks. To choose the df (knots) for temperature and lag, we used the Akaike information criterion (AIC) for quasi-Poisson models (Gasparrini et al. 2010; Peng et al. 2006). We found that using 5 df for temperature and 4 df for lag produced the best model fitting. We plotted the relative risks against temperature and lags to show the entire relationship between temperature and mortality. We also plotted the overall effect of temperature on morality summed over lag days.

Our initial analysis found that the temperature–mortality relationships were U-shaped, with potential cold and hot thresholds. Thus, we also used a double threshold–natural cubic spline DLNM, assuming the effect of cold temperature is linear below the cold threshold, whereas the effect of high temperature is linear above the hot threshold, and models the lag effects using a natural cubic spline with 4 df. Equation 1 was altered by modifying the β*T_i,l_* term into two linear threshold terms:

*Log* (*μ_t_*) = α + β*_c_TC_t,l_* + β*_H_TH_t,l_* + *COVs*, [2]

where *TC_t,l_* and *TH_t,l_* are matrices obtained by applying the double threshold–natural cubic spline DLNM to temperatures below the cold threshold and above the hot threshold respectively.

Temperature thresholds used in Equation 2 were determined by testing multiple thresholds. For example, for mean temperature, our initial analysis indicated that the potential cold threshold was within −5 to 5°C and the potential hot threshold was within 19–29°C. Hence we examined combinations of cold thresholds from −5.0 to 5.0°C (in 0.1°C gaps) and hot thresholds from 19.0 to 29.0°C (in 0.1°C gaps) to identify the combination that minimized the residual deviance. We then estimated the relative risks of mortality for a 1°C decrease in temperature below the cold threshold and a 1°C increase above the hot threshold.

The temperature–mortality relationship for combinations of temperature measures (maximum, mean, and minimum temperatures) and mortality categories (nonaccidental, cardiopulmonary, cardiovascular, and respiratory deaths) were each examined using the above steps. The AIC was used to choose the temperature measure that best predicted mortality.

Sensitivity analyses were performed by changing the window length in the case-crossover from calendar month to 30, 28, and 21 days to control for season and varying the maximum lags to 20 and 30 days for the DLNM.

All statistical tests were two-sided, and values of *p* < 0.05 were considered statistically significant. Spearman’s correlation coefficients were used to summarize the similarities in daily weather conditions. We used R software (version 2.12.1; R Development Core Team 2009) to fit all models, with its “dlnm” package to create the DLNM (Gasparrini and Armstrong 2011).

A detailed explanation of how to combine the case-crossover with DLNM is provided in the Supplemental Material, R code (http://dx.doi.org/10.1289/ehp.1103598).

## Results

The average daily maximum temperature was 19°C; mean temperature, 13°C; minimum temperature, 8°C; and relative humidity, 60%. The average daily mortality count for nonaccidental deaths was 56; cardiopulmonary deaths, 34; cardiovascular deaths, 30; and respiratory deaths, 4 (Table 1). The three temperature measures were strongly correlated with each other (Table 2).

**Table 1:** Summary statistics of daily weather conditions and mortality in Tianjin, China, 2005–2007.

Variable	Minimum	25%	Median	75%	Maximum	Mean ± SD
Temperature (°C)												
Maximum		–6		8		21		30		40		19 ± 12
Mean		–11		3		14		24		31		13 ± 11
Minimum		–14		–2		10		19		29		8 ± 11
Humidity (%)		13		46		61		74		97		60 ± 19
Death												
Nonaccidental		26		46		55		66		106		56 ± 14
Cardiopulmonary		13		27		33		40		77		34 ± 9
Cardiovascular		9		24		29		35		67		30 ± 8
Respiratory		0		3		4		6		15		4 ± 2
Influenza		0		0		0		0		2		0 ± 0.1
25% and 75% represent the 25th and 75th percentiles, respectively.												

**Table 2:** Spearman’s correlation coefficients between weather conditions in Tianjin, China, 2005–2007.

Temperature measure	Mean temperature	Minimum temperature	Humidity
Maximum temperature		0.98**		0.94**		0.16*
Mean temperature				0.98**		0.24*
Minimum temperature						0.32*
*p < 0.05. **p < 0.01.						

Mean temperature was generally associated with the lowest AIC values (i.e., had the best predictive ability for mortality) in Tianjin [see Supplemental Material, Table 1 (http://dx.doi.org/10.1289/ehp.1103598)]. The double threshold–natural cubic spline DLNM generally fit the data better than the natural cubic spline–natural cubic spline DLNM (see Supplemental Material, Table 1); therefore, we report results for associations with mean temperature only.

**Figure 1:**
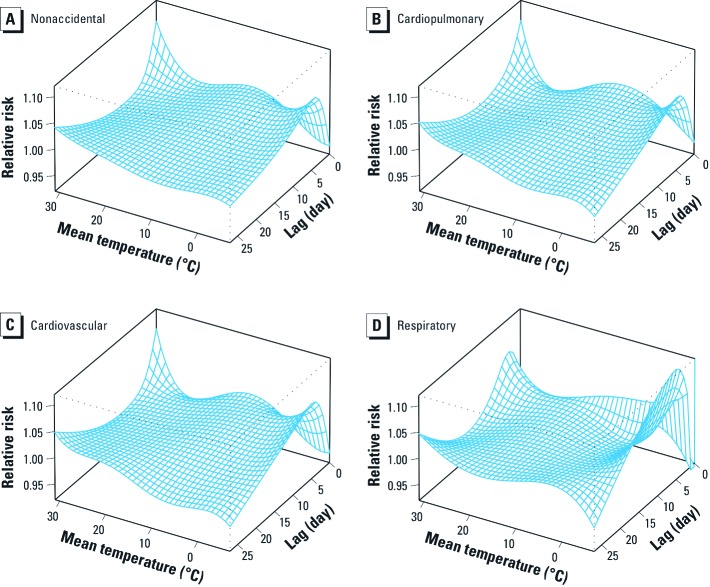
Relative risks of mortality types by mean temperature (°C), using a natural cubic spline–natural cubic spline DLNM with 5 df natural cubic spline for temperature and 4 df for lag. (*A*) Nonaccidental, (*B*) cardiopulmonary, (*C*) cardiovascular, and (*D*) respiratory mortality.

The three-dimensional plots show the entire surface between mean temperature and mortality categories at all lag days (Figure 1). The estimated effects of temperature were nonlinear for all mortality types, with higher relative risks at hot and cold temperatures. For example, extreme hot temperature (30°C) was positively associated with nonaccidental mortality on the current day, whereas extreme cold temperature (–6°C) significantly increased nonaccidental mortality after a 3-day lag. Neither hot effects (i.e., significant increases in mortality associated with hot temperatures) nor cold effects (i.e., significant increases in mortality associated with cold temperatures) were apparent after a 20-day lag, with relative risks close to 1 across the entire range of temperatures [see Supplemental Material, Figure 1(http://dx.doi.org/10.1289/ehp.1103598)].

**Figure 2:**
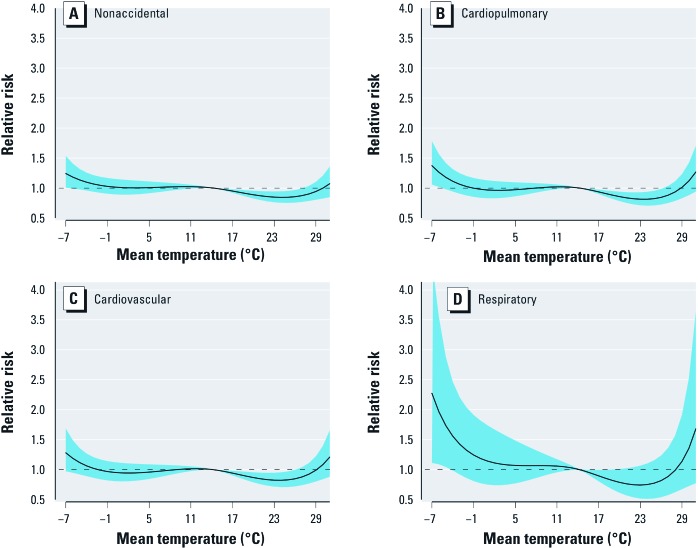
The estimated overall effects of mean temperature (°C) over 28 days on mortality types, using a natural cubic spline–natural cubic spline DLNM with 5 df natural cubic spline for temperature and 4 df for lag. (*A*) Nonaccidental, (*B*) cardiopulmonary, (*C*) cardiovascular, and (*D*) respiratory mortality. The black lines are the mean relative risks, and the blue regions are 95% CIs.

Figure 2 shows the estimated effect of mean temperature over 28 days on mortality. There were U-shaped relationships between mean temperature and all mortality types, with large comfortable temperature ranges where the relative risks of mortality were close to 1. The cold and hot thresholds (i.e., the temperatures below and above which estimates were constrained to be linear by the model and which do not necessarily coincide with temperatures associated with increased mortality by Equation 1) were 0.8°C and 24.9°C for nonaccidental mortality, 0.1°C and 25.3°C for cardiopulmonary mortality, 0.6°C and 25.1°C for cardiovascular mortality, and 0.7°C and 24.8°C for respiratory mortality.

**Figure 3:**
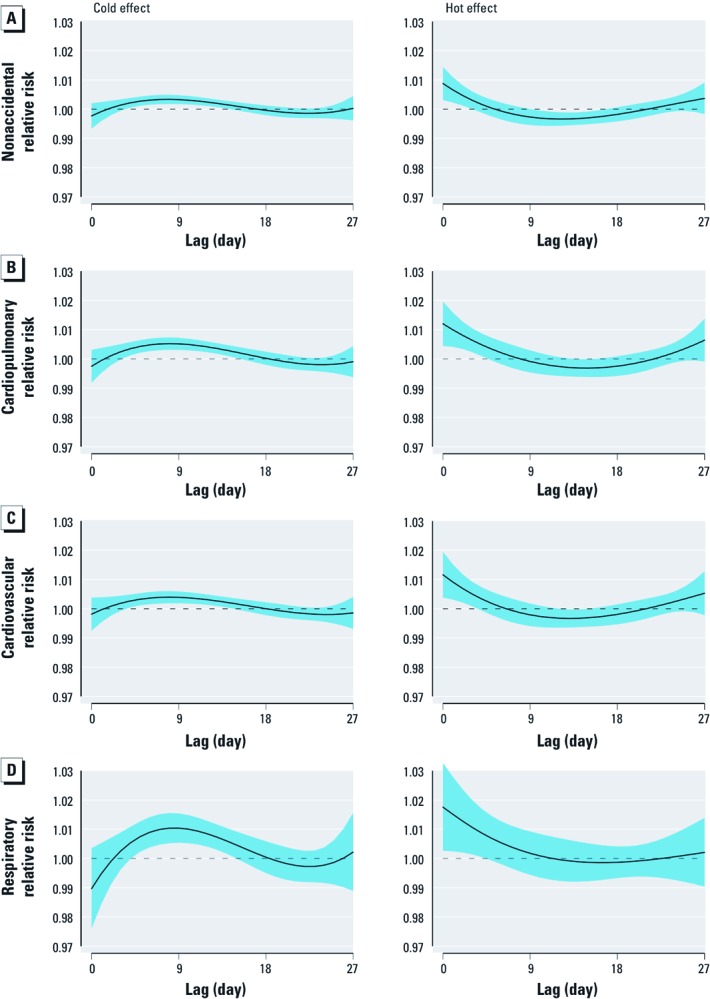
The estimated effects of a 1°C decrease in mean temperature below the cold threshold (left) and of a 1°C increase in mean temperature above the hot threshold (right) on mortality types over 27 days of lag, using a double threshold–natural cubic spline DLNM with 4 df natural cubic spline for lag. (*A*) Nonaccidental, (*B*) cardiopulmonary, (*C*) cardiovascular, and (*D*) respiratory mortality. The black lines are mean relative risks, and blue regions are 95% CIs. The cold and hot thresholds were 0.8°C and 24.9°C for nonaccidental mortality (*A*), 0.1°C and 25.3°C for cardiopulmonary mortality (*B*), 0.6°C and 25.1°C for cardiovascular mortality (*C*), 0.7°C and 24.8°C for respiratory mortality (*D*).

Significant cold effects appeared after a 3-day lag, whereas significant hot effects occurred within 0–3 days (Figure 3). Associations between cold and mortality lasted longer than associations with heat. Heat-related excesses of nonaccidental, cardiopulmonary, and cardiovascular mortality were followed by deficits in mortality, consistent with some mortality displacement caused by hot temperatures.

We calculated the overall effects of mean temperature on nonaccidental, cardiopulmonary, cardiovascular, and respiratory mortality along the lags (Table 3). For cold effects over lag 0–18 days, a 1°C decrease in mean temperature below the cold thresholds was associated with a 2.99% (95% confidence interval (CI): 0.85, 5.17) increase in nonaccidental deaths, 5.49% (95% CI: 2.29, 8.79) increase in cardiopulmonary deaths, 4.05% (95% CI: 1.14, 7.06) increase in cardiovascular deaths, and 9.25% (95% CI: 1.70, 17.37) increase in respiratory deaths. For hot effects over lag 0–2 days, a 1°C increase in mean temperature above the hot thresholds was associated with a 2.03% (95% CI: 0.70, 3.38) increase in nonaccidental deaths, 3.04% (95% CI: 1.24, 4.87) increase in cardiopulmonary deaths, 2.80% (95% CI: 0.95, 4.68) in cardiovascular deaths, and 3.36% (95% CI: –0.77, 7.67) increase in respiratory deaths. In general, cold effects of lag 0–27 days were greater than hot effects of lag 0–27 days except for respiratory mortality.

**Table 3:** The cumulative cold and hot effects of mean temperature on mortality categories along the lag days, using a double threshold–natural cubic spline DLNM with 4 df natural cubic spline for lag.

Percent increase in mortality (95% CI)						
Effect	Lag (days)	Nonaccidental	Cardiopulmonary	Cardiovascular	Respiratory
Cold effect*a*		0–2		–0.27 (–1.25, 0.72)		–0.19 (–1.49, 1.12)		–0.14 (–1.43, 1.17)		–1.65 (–4.75, 1.55)
		0–18		2.99 (0.85, 5.17)*		5.49 (2.29, 8.79)*		4.05 (1.14, 7.06)*		9.25 (1.70, 17.37)*
		0–27		2.13 (–0.44, 4.78)		4.16 (0.27, 8.21)*		2.66 (–0.86, 6.30)		7.99 (–1.08, 17.9)
Hot effect*b*		0–2		2.03 (0.70, 3.38)*		3.04 (1.24, 4.87)*		2.80 (0.95, 4.68)*		3.36 (–0.77, 7.67)
		0–18		–0.78 (–4.20, 2.77)		2.32 (–2.59, 7.49)		0.86 (–4.02, 5.98)		8.60 (–2.78, 21.31)
		0–27		0.31 (–3.48, 4.24)		3.83 (–1.75, 9.72)		2.47 (–2.99, 8.24)		8.79 (–3.62, 22.80)
**a**The percent increase in mortality for a 1°C of temperature decrease below the cold thresholds (0.8°C for nonaccidental, 0.1°C for cardiopulmonary, 0.6°C for cardiovascular, and 0.7°C respiratory mortality). **b**The percent increase in mortality for a 1°C of temperature increase above the hot thresholds (24.9°C for nonaccidental, 25.3°C for cardiopulmonary, 25.1°C for cardiovascular, and 24.8°C for respiratory mortality). *p < 0.05.										

*Sensitivity analysis.* We changed the window length of calendar month in the case-crossover to 30, 28, and 21 days, which gave similar results (data not shown). In addition, we changed the maximum lag to 20 and 30 days, which gave similar results (data not shown). Consequently, we believe that the models used in this study adequately captured the main effects of temperature on mortality.

## Discussion

*Temperature–mortality relationship.* The temperature–mortality relationship in Tianjin was U-shaped, with a large range of temperatures that were not associated with excess mortality. Significant associations between cold temperatures and mortality (cold effects) appeared after 3 days and lasted longer than the associations between high temperatures and mortality (hot effects), which were acute and of short duration. There was evidence of some mortality displacement due to effects of high temperatures on nonaccidental, cardiopulmonary, and cardiovascular deaths.

**Figure 4:**
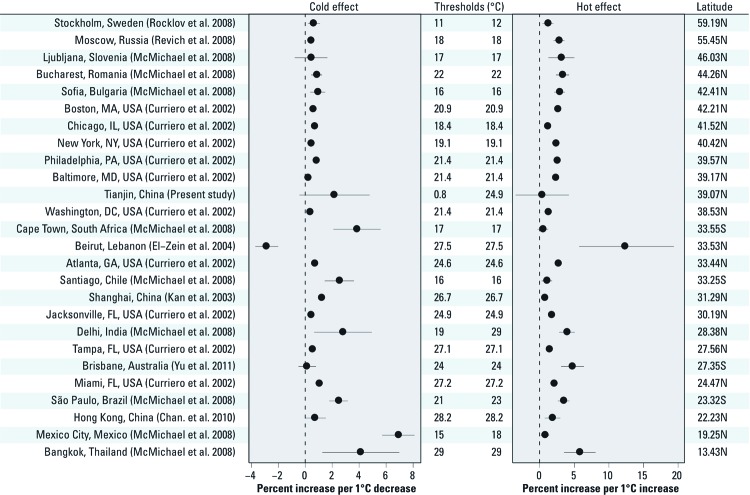
Comparison of the impacts of temperature on nonaccidental mortality in different populations ordered by latitude.

Many studies have examined the temperature–mortality relationship worldwide, but few studies are from China (Hajat and Kosatky 2010). We compared our results with studies that examined both cold and hot effects using mean temperature for nonaccidental mortality (Curriero et al. 2002; El-Zein et al. 2004; Revich and Shaposhnikov 2008; Rocklov and Forsberg 2008; Yu et al. 2011) (Figure 4). Results show that estimated temperature effects varied by region and population. Compared with populations living at similar latitudes, our results suggest a stronger cold effect and smaller hot effect. The reason might be that short lags were used in other studies, whereas we examined overall cold and hot effects of lag 0–27 days. Studies using short lags may have underestimated the cold effect: in our results, the estimated cold effect was delayed by 3 days and lasted for 10 days. Studies using short lags may overestimate the hot effect: in our results there was evidence of some mortality displacement, which can only be captured by using longer lags (Anderson and Bell 2009). Compared with other median or lower income populations (e.g., Bangkok, Mexico City, São Paulo, Delhi, Santiago, and Cape Town), Tianjin had lower cold and hot effects. The reason might be that people in Tianjin used protection measures in summer and winter (e.g., air conditioning and heating system) (McMichael et al. 2008).

We can compare our results with those from similar cities in China. Kan et al. (2003) found a V-shaped relationship between lag 0–2 days temperature and nonaccidental mortality in Shanghai, with an optimum temperature of 26.7°C. A 1°C decrease in temperature below 26.7°C increased nonaccidental mortality by 1.21%, while a 1°C increase in temperature above 26.7°C increased nonaccidental mortality by 0.73% (Kan et al. 2003). Liu et al. (2011) found both cold and hot temperatures were associated with increased cardiopulmonary mortality in Beijing, which has a climate similar to Tianjin’s. They also found an acute and short-term hot effect followed by some mortality displacement for cardiovascular mortality, consistent with our results.

An interesting finding is that the range of temperatures not associated with increased mortality is quite large in Tianjin, but extreme temperatures still had adverse effects on mortality. The exchange of heat between the body and surrounding temperature is regulated constantly by physiological control. Extreme high temperatures may cause a failure of thermoregulation, which may be impaired by dehydration, salt depletion, and increased surface blood circulation (Bouchama and Knochel 2002). Elevated blood viscosity, cholesterol levels, and sweating thresholds may also result in heat-related mortality (McGeehin and Mirabelli 2001). Cold temperatures increase the heart rate, peripheral vasoconstriction, blood pressure, blood cholesterol levels, plasma fibrinogen concentrations, and platelet viscosity (Ballester et al. 1997; Carder et al. 2005). In urban areas of Tianjin, 83% of houses had central heating in winter (Tianjin Statistic Bureau 2005), and 90% of homes had air conditioners (Tianjin Statistic Bureau 2004). However, although the majority of the urban population was potentially protected from the weather, extreme cold and hot days posed some increased risks.

We investigated lag effects over 28 days on mortality for both hot and cold days. In general, cold effects lasted about 10 days after the extreme cold period ended. Previous studies also reported similarly delayed cold effects on mortality (Anderson and Bell 2009; Goodman et al. 2004). The findings indicate that using short lags cannot completely capture the cold effect, so longer lags are required to examine the cold impact.

The hot effects were more acute and short-term. Studies have shown that hot temperatures induce an acute event in people with preexisting diseases (e.g., a previous myocardial infarction or stroke) and those who find it difficult to deal with heat (e.g., the elderly) (Muggeo and Hajat 2009). In people with congestive heart failure, the extra heat load may lead to fatal consequences (Näyhä 2005). The hot effect also led mortality displacement for nonaccidental, cardiopulmonary, and cardiovascular deaths—in agreement with studies conducted in Europe (Hajat et al. 2005; Pattenden et al. 2003) and the United States (Braga et al. 2001). Therefore, short lags cannot adequately be used to assess the hot effects, as the harvesting effects are ignored.

Studies of heat-related mortality have examined maximum, mean, or minimum temperatures, controlling for relative humidity (Anderson and Bell 2009). Other studies have used apparent temperature, the humidex, and temporal synoptic index (Zanobetti and Schwartz 2008). A large study of mortality in the United States found that the different measures of temperature had a similar ability to predict the impacts of temperature on mortality (Barnett et al. 2010). We found that maximum, mean, and minimum temperatures had similar predictive ability, probably because of their strong correlation. Overall, mean temperature performed best according to the AIC.

*Case-crossover design and DLNM.* Many models have been used to assess the impacts of temperature and air pollution on mortality and morbidity, such as descriptive (Reid et al. 2009), case-only (Schwartz 2005), case-crossover (Stafoggia et al. 2006), time-series (Hajat et al. 2002), and spatial analysis (Vaneckova et al. 2010). Generally, time-series and case-crossover designs are most commonly used in single or multiple locations over a time period. The main aim of both analyses is to examine associations between health and temperature after controlling for potential confounding factors such as secular trends and seasonal cycles (Basu et al. 2005). Using the case-crossover design, each subject is their own control, and so any confounding by fixed characteristics is removed. Another advantage of the case-crossover is that it controls for long-term and seasonal trends by design through short-interval strata (e.g., calendar month).

We compared the case-crossover design and a time-series design using a natural cubic spline with 7 df for time per year. The case-crossover design performed better than time-series analysis for this particular data based on AIC and residuals. However, we cannot conclude that the case-crossover is better than time-series for other data. We suggest checking the model fit and residuals when using case-crossover or time-series designs. In this study, we illustrated how to combine the DLNM with a case-crossover design to allow sophisticated nonlinear and delayed temperatures to be fitted using the case-crossover design.

One of the main advantages of DLNM is that it allows the model to contain detailed lag effects of exposure on response and provides the estimate of the overall effect that is adjusted for harvesting (Gasparrini et al. 2010). The DLNM can flexibly show different temperature–mortality relationships for lags using different smoothing functions. The DLNM can adequately model the main effects of temperature (Armstrong 2006).

There are also some issues in the selection of the DLNM, such as cross-basis type, maximum lag day, and degrees of freedom (knots and placement) for exposure and lag (Armstrong 2006; Gasparrini et al. 2010). Because the DLNM is combined with a regression model (e.g., Poisson regression), the residual deviance and autocorrelation plot, maximum likelihood, AIC, or Bayesian information criteria can be used to check the model. The options for the DLNM can be chosen according to the best model fit. Previous studies recommend choosing a DLNM that is easy to interpret from an epidemiological perspective (Armstrong 2006; Gasparrini et al. 2010). However, it is necessary to conduct sensitivity analyses to assess the key conclusions on model choice. In this study, we used AIC to select the degrees of freedom, and used residual deviance to choose both cold and hot thresholds, but used *a priori* arguments to choose cross-basis type and maximum lag day.

*Strengths and limitations.* This is the first study to give details on how to apply a DLNM in the case-crossover design and the first to assess the temperature–mortality relationship in Tianjin, China. We examined both cold and hot lag effects on four types of mortality and explored which temperature measure was the best predictor of mortality. Our findings can be used to promote capacity building for local response for extreme temperatures.

One limitation is that the data are from only one city, so it is difficult to generalize our results to other cities or to rural areas. We used the data on temperature and air pollution from fixed sites rather than individual exposure, so there may be some inevitable measurement error. The influence of ozone was not controlled for because data on ozone were unavailable. In previous research, hot effects were slightly reduced when ozone was controlled for, but cold effects were not changed (Anderson and Bell 2009). Some studies found a potential interaction between temperature and ozone (Ren et al. 2008). Further study needs to be conducted for this issue.

## Conclusions

DLNM can be applied in a case-crossover design so that the case-crossover can be used to examine sophisticated nonlinear and delayed effects of exposure (e.g., temperature or air pollution). Even though a relatively large temperature range was not associated with excess mortality, extreme cold and hot temperatures were associated with an increased risk of mortality in Tianjin, China. Cold temperatures had longer-lasting effects on mortality, whereas hot temperatures had acute and short-term effects.

## Supplemental Material

(319 KB) PDF
