# Supplementary material for: The Impact of Temperature on Mortality in Tianjin, China: A Case-Crossover Design with a Distributed Lag Nonlinear Model
Source: Environ Health Perspect. 2011 Aug 9;119(12):1719–25. doi: 10.1289/ehp.1103598 (PMC3261984; doi:10.1289/ehp.1103598)
Supplement: (319 KB) PDF [file ehp.1103598.s001.pdf]

Supplemental Material

The Impact of Temperature on Mortality in Tianjin, China: A Case–crossover Design with A Distributed Lag Non-linear Model

Yuming Guo[1], Adrian G Barnett[1], Xiaochuan Pan[2], Weiwei Yu[1], Shilu Tong[1]

[1] School of Public Health and Institute of Health and Biomedical Innovation, Queensland University of Technology, Brisbane, Australia.

[2] School of Public Health, Peking University, Beijing, China.

Table of contents

Supplemental Material, Table 1: Akaike information criteria (AIC) values for the relationship between temperature measures and mortality categories by DLNM type 3

Supplemental Material, Table 2: Cold and hot thresholds (°C) used by the "double threshold-natural cubic spline" 4

Supplemental Material, Figure 1: Relative risks by lag at specific mean temperatures (left) and relative risks by mean temperature at specific lags (right) for non-accidental mortality, using a "natural cubic spline-natural cubic spline" DLNM with 5 degrees of freedom for temperature and 4 degrees of freedom for lag. The reference temperature is 14 °C. 5

Supplemental Material, R code 6

References 11

Supplemental Material, Table 1: Akaike information criteria (AIC) values for the relationship between temperature measures and mortality categories by DLNM type

DLNM type	Temperature measure	AIC			
		Non-accidental	Cardiopulmonary	Cardiovascular	Respiratory
Natural cubic spline-natural cubic spline [a]	Maximum temperature	7494	6860	6679	4562
	Mean temperature	7472	6841	6658	4570
	Minimum temperature	7472	6840	6660	4580
Double threshold-natural cubic spline [b]	Maximum temperature	7488	6849	6662	4568
	Mean temperature	7473	6833	6653	4558
	Minimum temperature	7481	6845	6666	4556

[a] Using "natural cubic spline-natural cubic spline" DLNM with smoothing of 5 degrees of freedom for temperature and 4 degrees of freedom for lag;

[b] Using "double threshold-natural cubic spline" DLNM with smoothing of 4 degrees of freedom for lag; the cold and hot thresholds are shown in Supplemental Material, Table 2.

Supplemental Material, Table 2: Cold and hot thresholds (°C) used by the "double threshold-natural cubic spline"

Threshold type	Temperature measure	Mortality type			
		Non-accidental	Cardiopulmonary	Cardiovascular	Respiratory
Cold threshold (°C)	Maximum temperature	4.8	4.3	4.5	4.7
	Mean temperature	0.8	0.1	0.6	0.7
	Minimum temperature	–3.9	–4.1	–3.8	–3.5
Hot threshold (°C)	Maximum temperature	31.2	31.3	31.1	31.5
	Mean temperature	24.9	25.3	25.1	24.8
	Minimum temperature	20.1	22.0	21.9	21.6

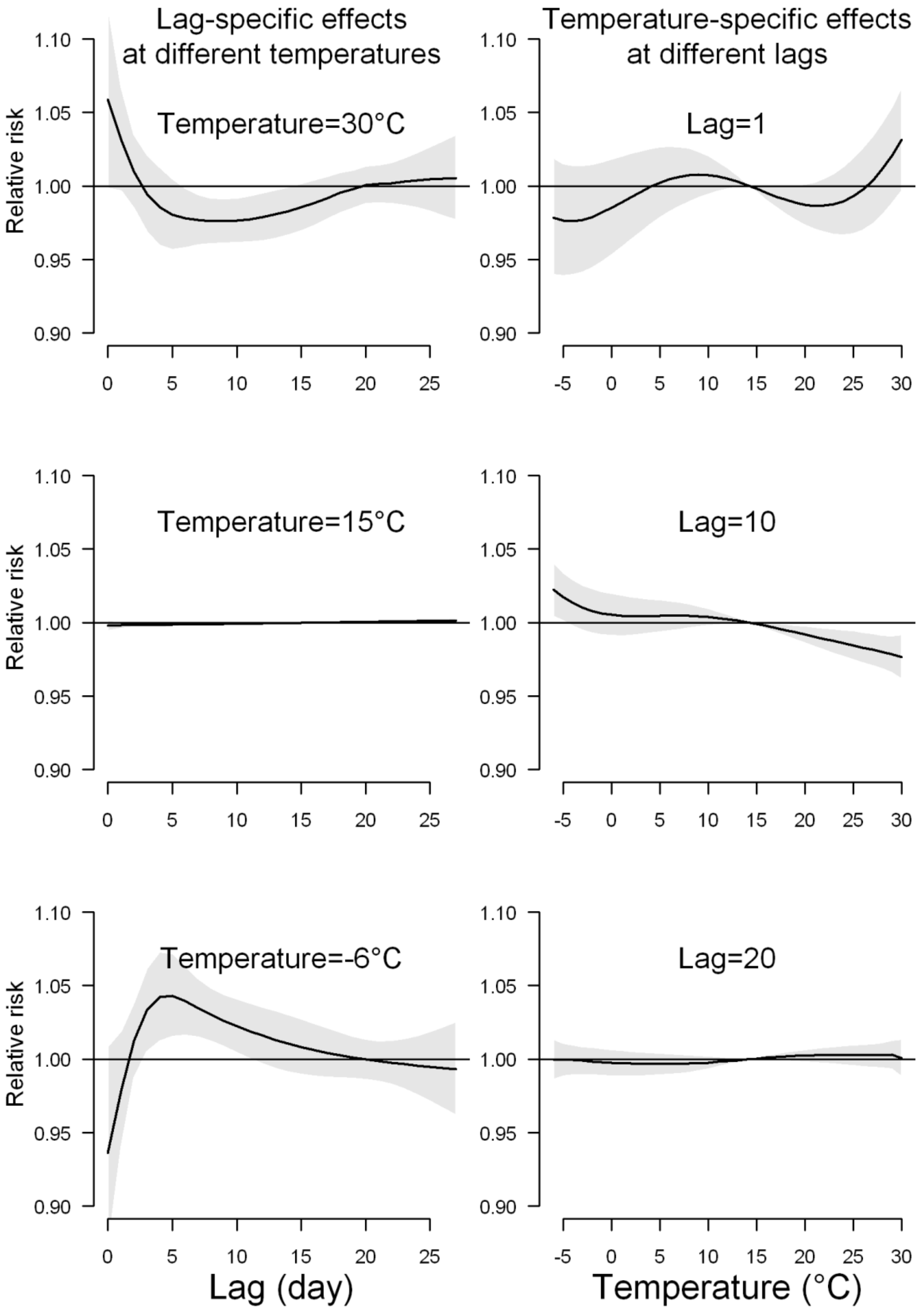

Supplemental Material, Figure 1: Relative risks by lag at specific mean temperatures (left) and relative risks by mean temperature at specific lags (right) for non-accidental mortality, using a "natural cubic spline-natural cubic spline" DLNM with 5 degrees of freedom for temperature and 4 degrees of freedom for lag. The reference temperature is 14 °C.

Supplemental Material, R code

As our data from Tianjin is not publicly available, we used data from Jersey city as an example. The data were from the National Morbidity, Mortality, and Air Pollution Study (NMMAPS) (Samet et al. 2000a; Samet et al. 2000b).

1. Load packages and prepare dataset:

```
>library(dlnm); library (NMMAPSlite)
>initDB()
>cities <- listCities()
# Jersey City: jers (city number 43)
>data <- readCity(cities[43], collapseAge = TRUE)
>data <- data[,c("city","date","death","inf","tmpd","rhum","so2mean","pm10trend")]
>data$temp <- (data$tmpd-32)*5/9  # Transfer temperature to Celsius
>data$time<-1:length(data[,1]) # Create time
>data$dow<-as.numeric(format(data$date,"%w")) # Create day of the week
>data$year<-as.numeric(format(data$date,"%Y")) # Create year
>data$month<-as.numeric(format(data$date,"%m")) # Create month
>data$strata<-data$year*100+data$month # Case-Control strata
```

2. Create Cross-basis matrix using "natural cubic spline-natural cubic spline" DLNM with 5 *df* for temperature and 4 *df* for lag

```
>range <- range(data$temp,na.rm=T)
>nknots<-4 # Number of knots for temperature
>nlagknots<-2 # Number of knots for lag
>ktemp <- range[1] + (range[2]-range[1])/(nknots+1)*1:nknots # Knots for temperature
>klag<-exp((log(27))/(nlagknots+2)*1:nlagknots) # Knots for lag
>basis.temp <- crossbasis(data$temp, vartype="ns", varknots=ktemp,
   cenvalue=median(data$temp,na.rm=T), lagtype="ns", lagknots=klag,maxlag=27)
```

3. Combine the case-crossover design with DLNM

```
>model.month <- glm(death ~ basis.temp + ns(rhum,df=3) + ns(pm10trend,df=3) +
                   ns(so2mean,df=3) + as.factor(I(inf>0)) + as.factor(strata)+as.factor(dow),
```

```
                    family=quasipoisson(), data)
```

4. Derive the predicted effects and standard errors for temperature and lags using DLNM

```
>pred.month <- crosspred(basis.temp, model.month, at=-16:32)
```

5. Plot 3D and overall effect graphics

```
> plot (pred.month,"3d",zlab="Relative Risk", r=90, d=0.3, col="red", xlab="Temperature",
   main="3D graphic for Jersey City", expand=0.6,lwd=0.5)
>plot(pred.month,"overall", xlab="Temperature (°C)", ylab=" Relative Risk ",
      main="Overall effect of temperature on mortality\n between 1987-2000 for Jersey City")
```

6. Determine the cold and hot thresholds (in °C) using "double threshold-natural cubic spline" DLNM

Based on the above 3D plot and overall effect plot, there are two potential thresholds for temperature. The cold threshold is somewhere between 0 to 8 °C, and hot threshold is somewhere between 19 to 26 °C. We used the following models to determine which combination of cold and hot thresholds gave the lowest residual deviance.

```
>cold.thr<-0:8  # In 1°C increments (In our study, we used 0.1°C increments)
>hot.thr<-19:26  # In 1°C increments (In our study, we used 0.1°C increments)
>deviance.matrix<-matrix(data = NA, nrow = length(cold.thr), ncol = length(hot.thr), byrow
                    = FALSE, dimnames = list(paste("cold.thr", cold.thr,sep="."),
                    paste("hot.thr", hot.thr,sep=".")))
>for (i in 1:length(cold.thr)){
  for (j in 1:length(hot.thr)){
  basis.try <- crossbasis(data$temp, vartype="dthr",varknots=c(cold.thr[i],hot.thr[j]),
               lagtype="ns", lagknots=klag, maxlag=27)
   model <- glm(death ~ basis.try + ns(rhum,df=3) + ns(pm10trend,df=3) + ns(so2mean,df=3)
          + as.factor(I(inf>0)) + as.factor(strata)+as.factor(dow), family=quasipoisson(), data)
   deviance.matrix[i,j]<-model$deviance
}
}
>row.col <- arrayInd(which.min(deviance.matrix), dim(deviance.matrix))
```

```
>rowname<-rownames(deviance.matrix)[row.col[,1]]
>colname<-colnames(deviance.matrix)[row.col[,2]]
>rowname;colname # Get the cold and hot thresholds
[1] "cold.thr.4" # The best cold threshold is 4°C
[1] "hot.thr.22" # The best hot threshold is 22 °C
```

7. Examine the cold (hot) effects below (above) the cold (hot)threshold using "Double threshold-natural cubic spline" DLNM

The cold threshold 4 °C and hot threshold 22 °C are used for a "Double threshold-natural cubic spline" DLNM.

```
>basis.cold.hot<- crossbasis(data$temp, vartype="dthr",varknots=c(4,22),
                      lagtype="ns", lagknots=klag, maxlag=27)
>model.cold.hot <- glm(death ~ basis.cold.hot + ns(rhum,df=3) +  ns(pm10trend,df=3) +
                      ns(so2mean,df=3) + as.factor(I(inf>0)) + as.factor(strata)+as.factor(dow),
                      family=quasipoisson(), data)
>cold.hot.pred <- crosspred(basis.cold.hot,model.cold.hot,at=-16:32)
> plot(cold.hot.pred,"3d",zlab="Relative Risk", r=90,d=0.3,col="red",xlab="Temperature",
main="\n3D graphic for Jersey City\nfor double threshold",expand=0.6,lwd=0.5) # 3D plot

>par(mfrow=c(2,1))
>plot(cold.hot.pred,"slices",var=c(3),main="Cold effect", xlab="", ylab=" Relative Risk ",
            ylim=range(0.99,1.01))
>plot(cold.hot.pred,"slices",var=c(23),main="Hot effect",xlab="Lag (day)",
                            ylab=" Relative Risk",   ylim=range(0.99,1.01))
```

8. Sensitivity analysis using 20 days as the maximum lag

```
> nlagknots<-2 # Number of knots for lag
> klag.20<-exp(log(20)/(nlagknots+2)*1:nlagknots) # Knots for lag
> basis.temp.20 <- crossbasis(data$temp, vartype="ns", varknots=ktemp,
       cenvalue=median(data$temp,na.rm=T), lagtype="ns",lagknots=klag.20,maxlag=20)
> model.month.20 <- glm(death ~ basis.temp.20 + ns(rhum,df=3) + ns(pm10trend,df=3) +
                      ns(so2mean,df=3) + as.factor(I(inf>0)) +as.factor(strata)+as.factor(dow),
                      family=quasipoisson(), data)
> pred.month.20 <- crosspred(basis.temp.20, model.month.20, at=-16:32)
```

```
> plot(pred.month.20,"overall", xlab="Temperature (°C)", ylab="Relative risk",
    main="Overall effect of temperature on mortality\n between 1987-2000 for Jersey
    City using maximum lag of 20 days")
```

9. Sensitivity analysis using 30 days as strata

```
>strata30<-floor((data$time-min(data$time))/30) # Create strata as 30 days
>model.strata30<- glm(death ~ basis.temp + ns(rhum,df=3) + ns(pm10trend,df=3) +
      ns(so2mean,df=3) + as.factor(I(inf>0)) +as.factor(strata30)+as.factor(dow),
            family=quasipoisson(), data)
>pred.strata30<- crosspred(basis.temp, model.strata30, at=-16:32, cumul=T)
>plot(pred.strata30,"overall", xlab="Temperature (°C)", ylab=" Relative risk ",
    main="Overall effect of temperature on mortality\n between 1987-2000 for Jersey
    City using 30 days as strata")
```

10. Comparison of time series and case–crossover design

```
# ignore humidity & pollution to remove influence of missing values
# case-crossover using calendar month as strata
>model.month <- glm(death ~ basis.temp + as.factor(I(inf>0))
      +as.factor(strata)+as.factor(dow), family=quasipoisson(), data)

# time series with 7 degrees of freedom for time per year
>model.ts <- glm(death ~ basis.temp + as.factor(I(inf>0)) +ns(time,98)+as.factor(dow),
            family=quasipoisson(), data)
```

# Plot the residual distribution

```
>par(mfrow=c(2,1))
> hist(resid(model.month),main="Residual distribution for case-crossover design\nusing
calendar month as strata",  xlim=range(-4,5),ylim=range(0,1100),xlab="Residuals",col="red",
font.lab=2,las=1)
>hist(resid(model.ts),main="Residual distribution for time series design\nusing 7 df for time
per year", xlim=range(-4,5),ylim=range(0,195),xlab="Residuals",col="red",font.lab=2,las=1)
>par(mfrow=c(1,1))
```

# Calculate AIC value for case-crossover

```
>AIC.cc<- -2*sum( dpois( model.month$y, model.month$fitted.values, log=TRUE))+
      2*summary(model.month)$df[3]*summary(model.month)$dispersion
   AIC.cc="26364.29"
```

# Calculate AIC value for time series

```
>AIC.ts <- -2*sum( dpois( model.ts $y, model.ts $fitted.values, log=TRUE))+
      2*summary(model.ts )$df[3]*summary(model.ts )$dispersion
  AIC.ts =" 26297.70"
```

For Jersey City, a time series design performs better than case-crossover as judged by the AIC. However, both designs give similar residuals. (For Tianjin, a case–crossover performed better than a time series according to both the AIC and residuals)

References

Samet JM, Dominici F, Zeger SL, Schwartz J, Dockery DW. 2000a. The National Morbidity, Mortality, and Air Pollution Study. Part I: Methods and methodologic issues. Res Rep Health Eff Inst(94 Pt 1): 5-14; discussion 75-84.

Samet JM, Zeger SL, Dominici F, Curriero F, Coursac I, Dockery DW, et al. 2000b. The National Morbidity, Mortality, and Air Pollution Study. Part II: Morbidity and mortality from air pollution in the United States. Res Rep Health Eff Inst 94(Pt 2): 5-70; discussion 71-79.
